# Variación biológica: un aspecto de la medicina de laboratorio aún en desarrollo

**DOI:** 10.1515/almed-2020-0003

**Published:** 2020-08-26

**Authors:** Callum G. Fraser

**Affiliations:** Centre for Research into Cancer Prevention and Screening, University of Dundee, Ninewells Hospital and Medical School, Dundee, Scotland

**Keywords:** especificaciones de la prestación analíticas, variación biológica, variación biológica interindividual, variación biológica intraindividual, valor de referencia del cambio

Hasta hace relativamente poco tiempo, el concepto de la variación biológica (VB) en el campo de la medicina laboratorio parecía ya desarrollado. Existe amplia evidencia de que, aunque algunos mesurandos presentan ritmos cíclicos, pudiendo estos ser diarios, mensuales o estacionales en naturaleza, la variabilidad en el tiempo de la mayoría de los analitos se puede considerar aleatoria [1]. Se ha revisado también la generación de datos numéricos de los componentes de la VB, entre los que se incluyen la VB intraindividual, esto es, la fluctuación media alrededor del punto homeostático (CV_I_), y la VB interindividual, es decir, las diferencias entre los puntos homeostáticos de diferentes individuos (CV_G_). Asimismo, la aplicación de los datos de CV_I_ y CV_G_, para establecer las especificaciones de prestación analítica para imprecisión, sesgo, error total analítico e incertidumbre de la medida, establecer el valor de referencia del cambio en resultados seriados de un individuo, evaluar la probabilidad de que un cambio en los resultados seriados sea significativo, calcular el índice de individualidad, o analizar la validez de los valores de referencia poblacionales, y realizar subdivisiones por edades y sexo, así como otros usos, está bien establecida y aceptada.

La aplicación de los datos de VB en el laboratorio de medicina ha sido posible gracias a la creación de una completa serie de bases de datos sobre componentes de VB, iniciada en 1997 y que integraba la información contenida en las publicaciones sobre VB. Inicialmente, se diseñó un sistema de puntuación para clasificar la robustez de los datos incluidos. Desde entonces, este increíblemente valioso recurso, que contaba con datos de 358 analitos [2] se fue actualizando bianualmente y publicándose en Internet hasta 2014. No obstante, comenzaron a surgir dudas sobre la precisión de algunas de las estimaciones incluidas en las bases de datos. Estas dudas se expusieron detalladamente en la 1^a^ Conferencia Estratégica de la Federación Europea de Laboratorio de Medicina (EFLM) *“Defining analytical prformance goals – 15 years after the Stockholm Conference”.* Estas aseveraciones fueron expuestas por Carobene que, aunque admitiendo que la utilidad de estas bases de datos era indudable, también parecía claro que podía verse afectada por algunos factores [3]. En aquella Conferencia se estableció la necesidad de crear una base de datos completa, proveniente de estudios adecuadamente ponderados, con suficiente potencia estadística y que proporcionaran valores numéricos sobre los componentes de VB, junto con sus intervalos de confianza. Además, la aplicación de una herramienta de lectura crítica permitiría la reevaluación de los datos existentes y aumentar la calidad de los futuros estudios. Del mismo modo, para satisfacer la necesidad de obtener estimados actuales, se podría crear un banco de muestras que, obtenidas adecuadamente, permitiría la generación de datos válidos de VB.

En los últimos cinco años, a través de varias iniciativas, la EFLM y otros grupos, en especial la Sociedad Española de Medicina de Laboratorio (SEQC^ML^), han establecido una serie de prerrequisitos necesarios para mejorar la generación y aplicación de datos sobre VB [4]. Entre los magníficos estudios realizados, se incluye el amplio Estudio Europeo sobre Variabilidad Biológica (EuBIVAS), del que ya se han obtenido datos de gran calidad sobre VB para una gran variedad de magnitudes, utilizando muestras recogidas en óptimas condiciones , minimizando las fuentes de variabilidad previas al análisis, mediante el empleo de métodos de medida actuales y meticulosos análisis estadísticos. Cabe señalar que, dado que sería imposible para todos los usuarios de datos de VB realizar sus propias estimaciones, al recomendarse el uso de las bases de datos, ahora se puede realizar una valoración crítica y metaanálisis de los estudios publicados sobre VB, empleando la *Biological Variation Data Critical Appraisal Checklist (BIVAC)* [5]. Actualmente, se pueden consultar los estimados globales de VB derivadas de la realización de un metanálisis, tras aplicar el BIVAC en página web de la EFLM creada por el Grupo de Trabajo de la EFLM (WG-BV) de VB y el Grupo de Trabajo de Base de Datos de Variabilidad Biológica (TG-BVD) [6]. Actualmente, esta base de datos incluye 488 publicaciones, 1.782 registros de datos sobre VB y datos sobre 143 magnitudes.

En este número de *Avances en Medicina de Laboratorio*, se incluye un estudio diseñado y realizado bajo altos estándares de calidad en el que se analizan los datos de VB de la glucosa y la HbA_1C_ [7]. En este estudio, se aplicó la *BIVAC* [5] a 40 artículos, 23 contenían datos de VB de la glucosa y 17 de HbA_1c_. El BIVAC contiene 14 preguntas, que clasifican la calidad de los artículos de VB en cuatro categorías, A, B, C o D, en orden de calidad descendente, tal como se indica en este estudio. Los estimados globales de VB (CV _I_ y CV_G_) se calcularon mediante un meta-análisis en el que se confería mayor peso a los artículos con mayor puntuación de calidad.

Cabe destacar que las nuevas estimaciones de VB de la glucosa y la HbA_1C_ son muy similares a las existentes en la anterior base de datos [2]. Podríamos plantearnos si esto es aplicable a otras magnitudes, ya que la nueva base de datos de la EFLM contiene datos únicamente de 143 magnitudes, mientras que la base de datos de 2014 incluye datos de 358 magnitudes. Quizá, esto signifique que los usuarios pueden seguir empleando la base de datos de 2014 con confianza, al menos hasta que la EFLM vaya ofreciendo nuevos datos obtenidos a partir de sus meta-análisis. Por ello, los posibles usuarios de datos de VB deberían consultar primero la base de datos de la EFLM [6] y, en caso de que no existan datos sobre el mesurando de interés, pueden recurrir a los ofrecidos en la base de datos de 2014, en caso de que estén disponibles.

Sin embargo, tal como se ha demostrado para otros mensurandos [3], solo un número reducido de los estudios analizados alcanzaron la máxima puntuación de calidad en la clasificación, y casi ninguno ofrecía datos sobre la estabilidad clínica de los sujetos. Así mismo, muy pocos verificaron la distribución normal de los datos, alrededor del 75% no evaluaron la homogeneidad de las varianzas, y menos de la mitad realizaron medidas duplicados para calcular la imprecisión (CV_A_). Si evaluamos los datos de VB obtenidos hasta la fecha de los mensurandos analizados en el estudio EuBIVAS, observamos que los nuevos estimados de CV_I_ y CV_G_ suelen ser bastante más estrechos que los de la base de datos de 2014. Presumiblemente, esto puede deberse a la meticulosa selección de sujetos aparentemente sanos, a la minimización de la variabilidad previa a la evaluación, al empleo de modernas técnicas de análisis con mayor especifidad, y la aplicación de técnicas estadísticas correctas excluyendo los valores aberrantes. Según Ricós et al.[7] , este hallazgo evidencia la necesidad de realizar más estudios de VB en las magnitudes empleadas para el diagnóstico y seguimiento de la diabetes mellitus, con el fin de mejorar las estimaciones de CV_I_ and CV_G,_ empleando métodos modernos y siguiendo la multitud de recomendaciones, prácticas clínicas y orientaciones promulgadas por la EFLM [4].

En otro de los artículos de este número, Díaz-Garzón et al. describen detalladamente los modelos actualmente disponibles para calcular los componentes de VB, expone sus ventajas e inconvenientes, y propone estrategias para la selección del método más adecuado para cada una de las aplicaciones de los datos de VB. Los modelos evaluados incluyen métodos directos para calcular los componentes de VB empleando el modelo ampliamente aplicado desarrollado por Fraser y Harris [8], recientemente revisados por la EFLM [4], modelos de efectos combinados, así como el más reciente y aún no muy extendido modelo bayesiano.

Dado que estos métodos para la realización de estudios sobre VB son complicados y requieren muchos recursos, especialmente si se llevan a cabo correctamente siguiendo las recomendaciones de la EFLM [4], existe un creciente interés en los métodos indirectos basados en estudios retrospectivos. Estos estudios denominados *“big data”* se realizan a partir de una gran cantidad de resultados sobre muestras de pacientes, que suelen estar disponibles en los sistemas informáticos de los laboratorios. A pesar de sus inconvenientes, claramente enumerados por Díaz-Garzón et al. [9], entre sus ventajas se encuentran el hecho de que se puede tener acceso fácilmente a variables como la edad, el género y la duración del estudio. Así mismo, el enorme número de sujetos incluidos aumenta la potencia estadística del estudio, el método resulta poco costoso, y no requiere estudios suplementarios como estudios experimentales o la aprobación de un comité ético. Este método ha sido analizado en un estudio reciente en el que se extrajeron pares de resultados consecutivos de una base general de datos sobre distintas patologías obtenidos en análisis bioquímicos y hematológicos de rutina. [10]. Cabría mencionar que este método arroja datos de CV_I_ coincidentes con los datos publicados para la mayoría de las 26 magnitudes analizadas. Las estimaciones mostraron un efecto mínimo de la edad, el género o el lapso de tiempo entre la obtención de muestras. De este modo, ampliando nuestra propuesta, los potenciales usuarios de datos de VB deberían consultar en primer lugar la base de datos de la EFLM [6] y, en caso de que esta no ofrezca datos sobre el mensurando en cuestión, se debería emplear la base de datos de 2014 [2], si estuvieran disponibles. Si no lo estuvieran, se podrían obtener los CV_I_ a partir de los resultados obtenidos en los pares de resultados de la base de datos de algún laboratorio, siguiendo las estrategias propuestas por Jones [10].

La VB es una cuestión aún en desarrollo en la medicina de laboratorio. Los dos artículos incluidos en este número [7], [9] contribuyen a mejorar la generación y aplicación de datos de VB y respaldan la adopción de un modelo jerárquico para la selección de datos sobre los componentes de la VB.
